# Applying Logistic Regression to Detect Differential Item Functioning in Multidimensional Data

**DOI:** 10.3389/fpsyg.2018.01302

**Published:** 2018-07-27

**Authors:** Hui-Fang Chen, Kuan-Yu Jin

**Affiliations:** ^1^Social and Behavioral Sciences, City University of Hong Kong, Kowloon, Hong Kong; ^2^Faculty of Education, The University of Hong Kong, Pokfulam, Hong Kong

**Keywords:** multidimensionality, differential item functioning, logistic regression, group impact, matching variables

## Abstract

Conventional differential item functioning (DIF) approaches such as logistic regression (LR) often assume unidimensionality of a scale and match participants in the reference and focal groups based on total scores. However, many educational and psychological assessments are multidimensional by design, and a matching variable using total scores that does not reflect the test structure may not be good practice in multidimensional items for DIF detection. We propose the use of all subscores of a scale in LR and compare its performance with alternative matching methods, including the use of total score and individual subscores. We focused on uniform DIF situation in which 250, 500, or 1,000 participants in each group answered 21 items reflecting two dimensions, and the 21st item was the studied item. Five factors were manipulated in the study: (a) the test structure, (b) numbers of cross-loaded items, (c) group differences in latent abilities, (d) the magnitude of DIF, and (e) group sample size. The results showed that, when the studied item measured a single domain, the conventional LR incorporating total scores as a matching variable yielded inflated false positive rates (FPRs) when two groups differed in one latent ability. The situation worsened when one group had a higher ability in one domain and lower ability in another. The LR using a single subscore as the matching variable performed well in terms of FPRs and true positive rates (TPRs) when two groups did not differ in either one latent ability or differed in one latent ability. However, this approach yielded inflated FPRs when two groups differed in two latent abilities. The proposed LR using two subscores yielded well-controlled FPRs across all conditions and yielded the highest TPRs. When the studied item measured two domains, the use of either the total score or two subscores worked well in the control of FPRs and yielded similar TPRs across conditions, whereas the use of a single subscore resulted in inflated FPRs when two groups differed in one or two latent abilities. In conclusion, we recommend the use of multiple subscores to match subjects in DIF detection for multidimensional data.

## Introduction

Differential item functioning (DIF) is commonly assessed to examine the prerequisite of test fairness (Stark et al., 2006) and has become routine practice in large-scale educational assessments such as the Trends in Mathematics and Science Study (TIMSS) and the Programme for International Student Assessment (PISA). The presence of DIF indicates an unequal probability that two groups will accurately answer or endorse an item, where participants in both groups have the same levels of ability.

Multidimensionality is common in educational and psychological tests. All abilities measured by these tests are intentional, and studies that use exploratory factor analysis often report an underlying multidimensional construct in the data (Mazor et al., 1995; Adams et al., 1997). Multidimensionality data can be categorized into two types: between-item and within-item multidimensionality (Wang et al., 1997). A test comprising several subtests that measure distinct latent abilities, and in which test items reflect a single construct, represents a simple test structure and yields between-item multidimensional data. The TerraNova Multiple Assessment Test and the Minnesota Multiphasic Personality Inventory are two representative examples of between-multidimensional tests. On the other hand, within-item multidimensionality, or complex structure, refers to items within a test measuring two or more dimensions and providing information for at least two domains of a scale. For instance, in Teacher Education and Development Study in Mathematics, 72 items measure a unidimensional construct of mathematics content knowledge, and 32 items measure both mathematics content knowledge and mathematics pedagogical content knowledge (Blömeke et al., 2011), yielding within-item multidimensional data. Although within-item multidimensionality commonly occurs in empirical studies, researchers often eliminate these cross-loaded items in subsequent analyses, thereby implying that certain information may be missed.

A common practice in DIF research is to use a unidimensional calibration to yield a reference composite of the underlying multiple dimensions for the entire test (Mazor et al., 1998). The unidimensional approach of DIF detection is feasible for analyzing between-multidimensional data because a between-multidimensional scale can be decomposed into multiple unidimensional subscales. Given the assumption of unidimensionality of a scale, when an item measures at least one secondary dimension (in addition to the primary dimension the measurement is intended to measure) and two groups of examinees differ in their underlying ability distribution of the secondary dimensions, DIF occurs (Ackerman, 1992; Bolt and Stout, 1996; Roussos and Stout, 1996; Walker and Gocer Sahin, 2017). Using the multidimensional item response theory (IRT; Shealy and Stout, 1993; Walker and Gocer Sahin, 2017), the expected difference of scores between the reference (R) and focal (F) groups for an item under consideration can be expressed in the following manner:

(1) $$\begin{array}{l} \begin{array}{c} E_{\text{R}}(\eta |\theta )-E_{\text{F}}(\eta |\theta )=\left(\mu_{\eta_{\text{R}}}-\mu_{\eta_{\text{F}}})+\theta (\rho_{\text{R}}\frac{\sigma_{\eta_{\text{R}}}}{\sigma_{\theta_{\text{R}}}}-\rho_{\text{F}}\frac{\sigma_{\eta_{\text{F}}}}{\sigma_{\theta_{\text{F}}}}\right) \end{array}\\ \begin{array}{c} \;+(\mu_{\theta_{\text{F}}}\rho_{\text{F}}\frac{\sigma_{\eta_{\text{F}}}}{\sigma_{\theta_{\text{F}}}}-\mu_{\theta_{\text{R}}}\rho_{\text{R}}\frac{\sigma_{\eta_{\text{R}}}}{\sigma_{\theta_{\text{R}}}}), \end{array} \end{array}$$

where μ indicates the mean ability, σ is the standard deviation (SD), and ρ is the correlation between the primary (θ) and secondary abilities (η). The expected difference, a plausible DIF, can vary conditionally on the two distributions of θ. When two groups have identical SDs of θ (σ_θ_R__ = σ_θ_F__) and of η (σ_η_R__ = σ_η_F__) as well as the same relationship between two latent abilities (ρ_R_ = ρ_F_), Equation (1) becomes

(2) $$\begin{array}{r} E_{\text{R}}\left(\eta |\theta \right)-E_{\text{F}}\left(\eta |\theta \right)=\left(\mu_{\eta_{\text{R}}}-\mu_{\eta_{\text{F}}}\right)-\rho \left(\mu_{\theta_{\text{R}}}-\mu_{\theta_{\text{F}}}\right). \end{array}$$

Equation (2) illustrates the presence of DIF depending on the magnitude of the group difference in the averages of two latent abilities and the correlation between two dimensions. When μ_η_R__ ≠ μ_η_F__, DIF is most likely to occur when ρ(μ_θ_R__ − μ_θ_F__) has a different sign and magnitude from μ_η_R__ − μ_η_F__ (Ackerman, 1992; Roussos and Stout, 1996). DIF may occur when the correlations between θ and η are not identical for the two groups and the two groups differ in the means of θ. In addition, even when θ is held constant, DIF may occur due to the difference between individuals' secondary abilities from two different groups.

Equation (2) also suggests that DIF is less likely to occur when ρ(μ_θ_R__ − μ_θ_F__) has the same sign as μ_η_R__ − μ_η_F__. Specifically, when two groups have identical ability distributions of η under either condition of (1) a zero correlation between θ and η, or (2) an identical distribution of θ for the two groups, DIF will not occur.

In conclusion, Equations (1) and (2) imply that, when assessing DIF in a multidimensional scale, all the measured latent traits should be jointly considered (Ackerman, 1992; Roussos and Stout, 1996; Yao and Li, 2015); otherwise, the conclusion of measurement invariance could be biased. Specifically, when a DIF-free item measures a second intended-to-be-measured domain, but the multidimensional structure is ignored in the matching variable, this item would be mistakenly classified as DIF. On the other hand, regardless of the dimensionality of the studied item, when certain anchored items are within-item multidimensional but are ignored, the matching variable is contaminated and then leads to biased DIF detection.

Conventional DIF practices assume unidimensionality of a scale and use the total score to match respondents from different groups on a common metric. For a test developed to measure more than one latent trait, however, the total score might not provide sufficient information to describe the multidimensional distributions of latent traits, unless the latent traits are highly correlated. When a total score is composed of two poorly correlated subscores, the relationship between the total score and either subscore would be severely attenuated, which could reduce the representativeness of a matching variable and, in turn, decrease the accuracy of the DIF assessment.

In addition, even when two groups show two distinct multidimensional ability distributions, the interpretation of the total score may differ for the two groups across all ability levels. Suppose that examinees A and B differ in the two-dimensional traits, their abilities are denoted as (−1, 1) and (1, −1), respectively, and their overall performance (total score) is estimated to be equal. In reality, examinee A will have a lower probability than examinee B of accurately answering items that measure the first domain and will show a higher probability of correctly answering items that reflect the second domain. Thus, if total scores serve as a matching variable to place the two participants from different groups on the same scale, then uncontrolled between-group ability differences between these two domains may yield inaccurate DIF detection (Mazor et al., 1995). Yao and Li (2010, 2015) found that fitting a unidimensional IRT to multidimensional data yielded an inflated false positive rate (FPR), where the item was detected as DIF when two groups have different abilities and suggested the use of multidimensional IRT. Therefore, it is concluded that the necessity of a matching variable could accurately reflect test dimensions.

Previous studies have suggested the use of a multidimensional framework (e.g., Zwick and Ercikan, 1989; Clauser et al., 1991; Mazor et al., 1998; Walker and Gocer Sahin, 2017) for DIF detection by using a matching alternative, other than total scores, in the Mantel-Haenszel (MH) approach (Mantel and Haenszel, 1959; Holland and Thayer, 1988), the simultaneous item bias test (SIBTEST, Shealy and Stout, 1993), and the logistic regression (LR) approach (Swaminathan and Rogers, 1990; Rogers and Swaminathan, 1993). For example, Ackerman and Evans (1994) used an arbitrary 64 units in the two-dimensional latent trait space in the MH approach as an alternative and compared its performance with that of conventional matching variables (total scores). In their study, two-dimensional data were generated for 30 items, in which two items measured a single domain, while others reflected different degrees of two domains. Both the MH and SIBTEST procedures using total scores as a matching variable yielded severely inflated type I errors, but inflation was eliminated when multiple subdomain scores were used as a matching variable. Studies also found that items with higher loadings from one specific domain were consistently identified as DIF items when the subdomain score of the other domain was used for matching (Ackerman and Evans, 1994; Walker and Gocer Sahin, 2017). Similar results have been found in the LR procedure as well (Zwick and Ercikan, 1989; Mazor et al., 1998). The LR approach is generally recommended because it is readily expandable to include more than one ability estimate for matching, whereas the MH approach becomes increasingly cumbersome and arbitrary when handling two or more ability estimates (Mazor et al., 1998).

Although the above findings are strongly supportive of the use of a matching variable that more closely approximates the underlying test structure, several factors have not been carefully considered in these studies.

The first concern is the composite of a matching alternative used in these studies. To assess DIF in multidimensional data, Ackerman and Evans (1994) used true values of different latent abilities to match participants. This approach is not applicable in practice, however, because one would never know a participant's “true” ability beforehand. Some studies (e.g., Mazor et al., 1995; Clauser et al., 1996) have suggested the use of all summed scores of subtests as matching variables and have assumed that all items were designed to assess a single domain (i.e., between-item multidimensionality). However, the tests in these studies were within-item multidimensional, and cross-loaded items were not counted in both domains but in one only. In other words, these within-dimensional items were not accurately counted in previous studies. Therefore, their findings did not provide strong evidence for the use of alternatives in practice. How the dimensionality of the test structure interacts with matching variables in DIF detection remains unclear.

The second concern is impact on the mean ability in terms of group differences in the mean ability. Walker and Gocer Sahin (2017) reported that, when conditioning on the primary domain, a small mean difference in the secondary dimension could cause DIF independently of the relations between or among domains. Such findings may suggest that DIF will not occur when conditioning on both the primary and secondary dimensions simultaneously. In other words, if a matching variable (or variables) could accurately reflect the test structure and match participants on the same metric in the primary and secondary domains, it is possible that the items are not mistakenly identified as DIF items. Hence, the use of different matching variables could result in different conclusions in DIF detection. These inferences must be investigated before any suggestions for practical use can be made.

To the best of our knowledge, most researchers have studied DIF issues in unidimensional data. For between-item multidimensional data, items are grouped as multiple unidimensional subscales, and DIF analyses are conducted for each subscale. Existing literature has not adequately addressed within-item multidimensional data in DIF analyses. The present study examines (1) the impact of dimensionality in DIF detection when tests are designed to measure two domains and (2) the impact of group mean differences in one or both latent abilities. The calculations for two subtest scores were revised to accurately reflect the underlying test structure, and the performance of this method was compared to that of other matching alternatives. Factors such as test structure (both within- and between-multidimensionality), group means of latent abilities, and percentage of cross-loaded items in a test were manipulated to account for these factors' influence on and interactions with the dimensionality of a matching variable. Models that incorporated a matching variable closely approaching that of the test structure were expected to yield more satisfactory results than models that failed to account for underlying domains.

## Logistic regression (LR) for detecting differential item functioning

LR is a viable and flexible procedure for detecting DIF that does not require specific forms of item response function or large sample sizes (Narayanan and Swaminathan, 1994). It also demonstrates computational simplicity and is easily implementable using commercial software (e.g., SPSS, SAS, or STATA) or free software (e.g., R) without additional effort or knowledge. Mazor et al. (1995) have suggested that the LR procedure can readily be expanded to explicitly include two or more domains and is therefore particularly suitable for the analysis of multidimensional data. Thus, the present study only focuses on the impact of the abovementioned factors on LR in DIF detections. The LR procedure can be denoted in the following manner:

(3) $$\begin{array}{r} \log\left[\frac{P\left(Y=1|G_{i},X_{i}\right)}{P\left(Y=0|G_{i},X_{i}\right)}\right]=\beta_{0}+\beta_{1}X_{i}+\beta_{2}G_{i}+\beta_{3}G_{i}X_{i}. \end{array}$$

Equation (3) states that a person *i* belonging to group *G* has a probability of accurately answering a question against a probability of answering incorrectly; *X* indicates the ability level measured by a test (a matching variable), which is usually the observed total test score; and *GX* is the interaction of the grouping membership and the test score. If β_2_ ≠ 0 and β_3_ = 0, then an item shows uniform DIF, thereby implying that item difficulty parameters differ among groups. If β_3_ ≠ 0, then non-uniform DIF has occurred, and item discrimination parameters differ across groups. Because uniform DIF is a more commonly investigated phenomenon, the present study only examines uniform DIF. Equation (3) could be extended to incorporate the scores of multiple dimensions in the LR procedure, in the following manner:

(4) $$\begin{array}{l} \log\left[\frac{P\left(Y=1|G_{i},{X_{1i},\ldots ,X}_{ni}\right)}{P\left(Y=0|G_{i},{X_{1i},\ldots ,X}_{ni}\right)}\right]=\beta_{0}+\sum_{n=1}^{N}\beta_{1n}X_{ni}\\ \;+\beta_{2}G_{i}, \end{array}$$

where *X*_*ni*_ is the sum of scores in the *n*th dimension for person *i* (i.e., the *n*th subscore of the test).

**Figure 1:**
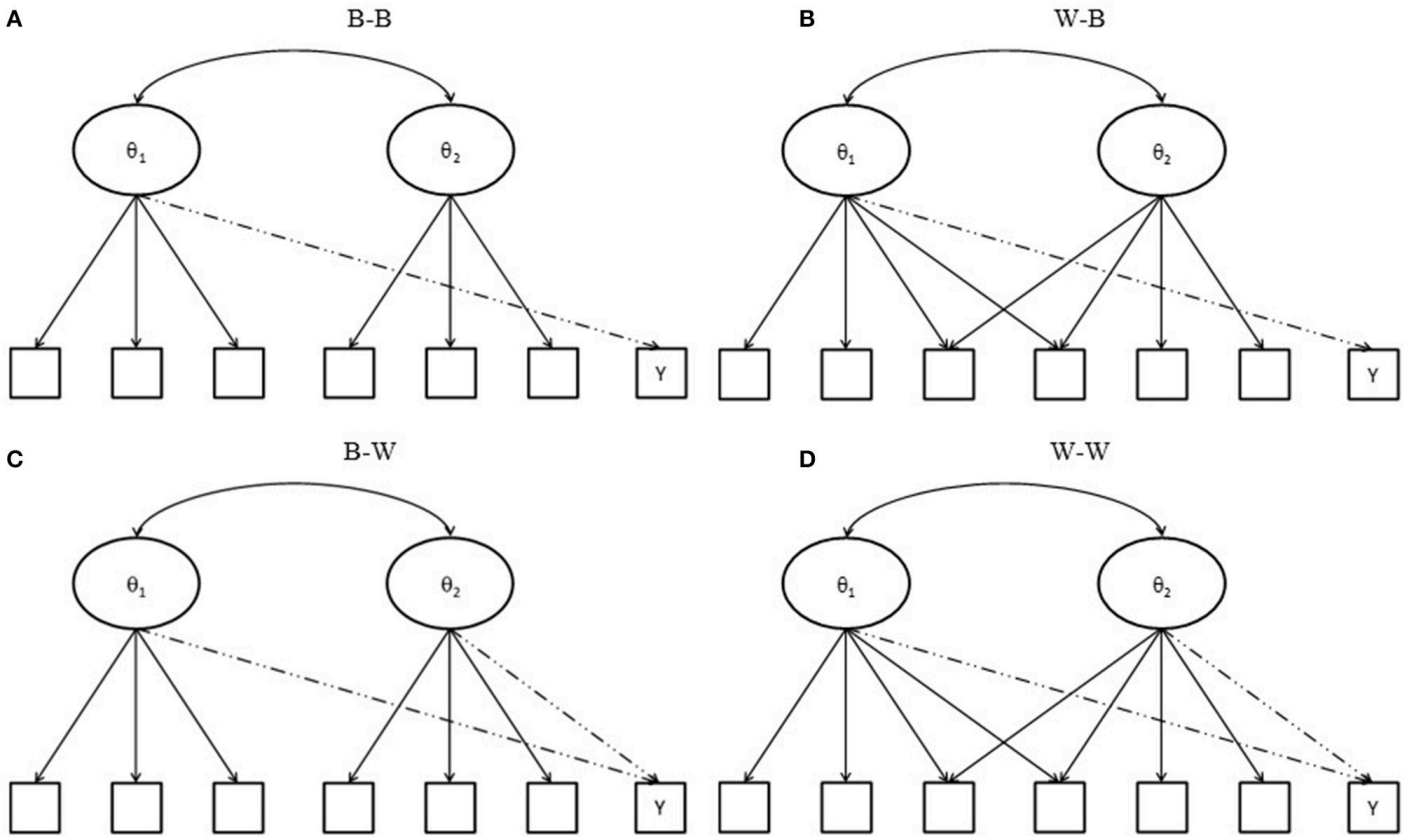
Test structure of a matching variable and an item under consideration. Y indicates an item under consideration; **(A)** B-B indicates between-item multidimensionality of a matching variable with a between-item multidimensional item under consideration; **(B)** W-B indicates within-item multidimensionality of a matching variable with a between-item multidimensional item under consideration; **(C)** B-W indicates between-item multidimensionality of a matching variable with a with-item multidimensional item under consideration; and **(D)** W-W indicates within-item multidimensionality of a matching variable with a within-item multidimensional item under consideration.

Suppose that a test comprises two subscales, and that some items are designed to measure two domains. When both between- and within-item multidimensionality might occur in the matching variable and the item under consideration, four conditions exist, as depicted in Figure 1. The first condition is a between-between condition (named B-B), in which all items measure a single domain; the second is within-between (named W-B), in which some items of the matching variable measure two domains, while others and the items under consideration reflect a single domain. The third is between-within (named B-W), where all but the item under consideration measure a single domain; the last is within-within (W-W), where some items of a matching variable and the item under consideration measure two domains and the remaining items measure one domain only. Note that when within-multidimensionality occurs in the matching variable, any items that measure two domains should be double counted in the summed scores of the first and second domains because they simultaneously measure two latent abilities. Consequently, four models can be used for DIF assessment, including model 1 (using a total score, in other words, the sum score of domain 1 plus the sum score of domain 2), model 2a (using the sum score of domain 1), model 2b (using the sum score of domain 2), and model 3 (using the two subscores, the sum score of domain 1 and the sum score of domain 2, together).

In cases where the tested item measures domain 1 ability (e.g., B-B and W-B conditions), models 1, 2a, and 3 will yield accurate DIF detection when no impacts have occurred in the primary and secondary domains. Because impacts and the correlation between two domains both increase, model 3 becomes the only model to use an effective matching variable to provide sufficient information that can accurately locate participants in the same scale for comparison. It will yield satisfactory results. The performances of models 1 and 2a, however, will be influenced by the number of cross-loaded items within the test as well as by the relationships among subdomains; they will yield inflated type I error rates. On the other hand, when the tested item measures both domain 1 and domain 2 abilities (e.g., B-W and W-W conditions), the use of total scores or using two subdomain scores simultaneously will accurately match participants in the primary and secondary domains and yield more accurate DIF detection; on the other hand, since models 2a and 2b could not provide sufficient information of dimensionality, they would perform poorly. A series of simulations was conducted to test the above hypotheses.

## Simulation study design

Several simulation studies were conducted to detect uniform DIF in two-dimensional data. Item responses were simulated according to the multidimensional three-parameter logistic model in uniform DIF setting, in the following manner:

(5) $$\begin{array}{r} P\left(Y_{ij}=1\right)=\gamma_{j}+\left(1-\gamma_{j}\right)\times \frac{\exp\left(\sum_{d=1}^{D}\alpha_{jd}\theta_{id}-\beta_{j}\right)}{1+\exp\left(\sum_{d=1}^{D}\alpha_{jd}\theta_{id}-\beta_{j}\right)}, \end{array}$$

where θ_*id*_ is the *d*th ability of person *i*, α_*jd*_ is the slope of dimension *d* of item *j*, and β_*j*_ and γ_*j*_ are the difficulty and guessing parameters, respectively, of item *j*. Twenty-one items were designed to measure (1) either one ability only or (2) both abilities. Specifically, the first 20 items were DIF-free items and were, therefore, used as the matching variable (termed anchor items), and the final item was under suspicion. The first half of the anchor items was designed to measure the first domain, and the second half to measure the second domain. Under conditions in which 4 anchor items measured both domains, the first 8 anchor items measured the first domain, items 9–12 measured both domains, and items 13–20 reflected the second domain only. When there were 8 cross-loaded items, items 1–6 assessed the first domain, items 7–14 measured both, and items 15–20 measured the second domain. Difficulty parameters were generated from *N*(0,1). Discrimination parameters followed a log-normal distribution, with a mean of 0 and variance of 0.3, and all guessing parameters were fixed at 0.2. When there was no group difference (termed “impact” in DIF detection) in both latent abilities, the ability of the two domains followed a bivariate normal distribution with a mean vector of 0 and variance of 1, and the covariance between two domains was set at 0.2 for both the focal and reference groups. When impact existed, the mean vector was manipulated (see point 5 for details). Five independent factors were manipulated:

- The number of dimensions measured by the item under consideration (one or two domains);
- Numbers of cross-loaded anchor items (0, 4, or 8 items);
- The magnitudes of DIF (the item difficulty for the focal group was 0.3 or 0.5 higher than those for the focal group to present medium and large DF sizes, respectively; Gonzalez-Roma et al., 2006);
- The sample size in each group of examinees (250, 500, or 1,000); and
- Impact at (0, 0), (0.5, 0), or (0.5, −0.5) for the focal and reference groups in the primary and secondary domains, respectively, by manipulating the deviation from the mean of the mean vector.

A total of 1,000 replications were conducted under each condition. Any FPRs and true positive rates (TPRs) of items under consideration were computed. The nominal alpha level was 0.05. When the item under consideration measured one domain (domain 1), the performance of models 1, 2a, and 3 was compared; when the tested item measured two domains, the performance of the four alternatives was evaluated.

## Results

**Figure 2:**
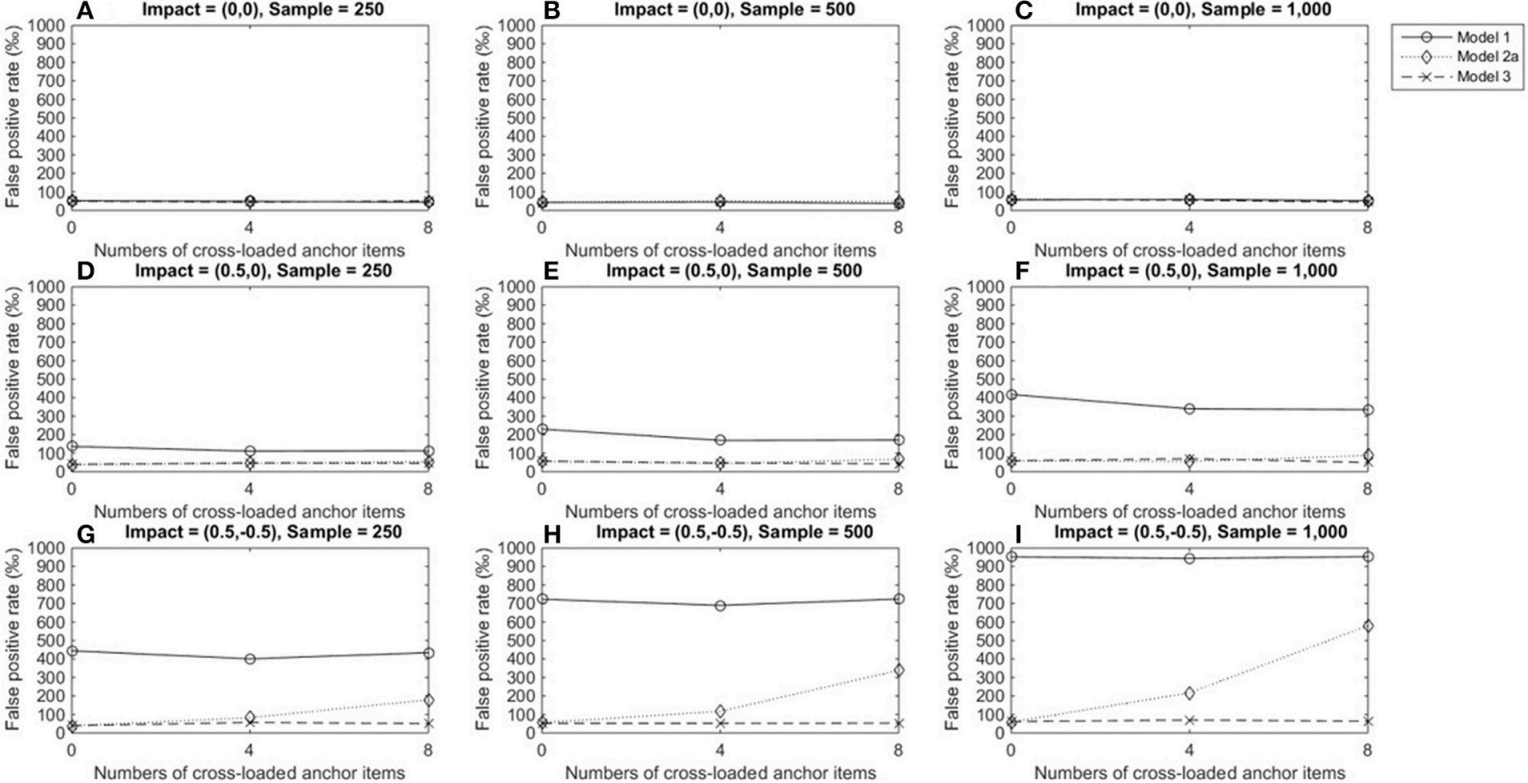
False positive rates when the tested item measured one single domain. For model 1, total scores served as a matching variable; for model 2a, the subset score of domain 1 was used to match participants; model 3 used two subset scores as a matching variable. Model 2b was excluded because the tested item did not measure the second domain. **(A)** Impact = (0,0), Sample = 250; **(B)** Impact = (0,0), Sample = 500; **(C)** Impact = (0,0), Sample = 1,000; **(D)** Impact = (0.5,0), Sample = 250; **(E)** Impact = (0.5,0), Sample = 500; **(F)** Impact = (0.5,0), Sample = 1,000; **(G)** Impact = (0.5, −0.5), Sample = 250; **(H)** Impact = (0.5, −0.5), Sample = 500; **(I)** Impact = (0.5, −0.5), Sample = 1,000.

Figure 2 depicts the FPRs when the item under consideration measured a single domain (domain 1); Figures 2A–C show that the three alternatives yielded satisfactory FPRs of 0.05 when the reference and focal groups had identical ability distributions in the primary and secondary domains and their performance was not influenced by the number of cross-loaded anchor items or group size. Figures 2D–F show that when the two groups differed in the primary dimension, models 2a and 3 showed good control of FPRs, but model 1 yielded severely inflated FPRs across conditions and the inflation increased as group sizes increased. When the two groups differed in both the primary and secondary domains, model 3 still showed satisfactory FPRs across conditions (Figures 2G–I). However, Model 2a yielded inflated FPRs when anchor items measured two dimensions, and the inflation increased as the number of cross-loaded anchor items increased. Model 1 performed even more poorly: FPRs were severely inflated when impacts occurred in both dimensions, regardless of the number of cross-loaded anchor items, and inflation worsened as group sizes increased.

**Figure 3:**
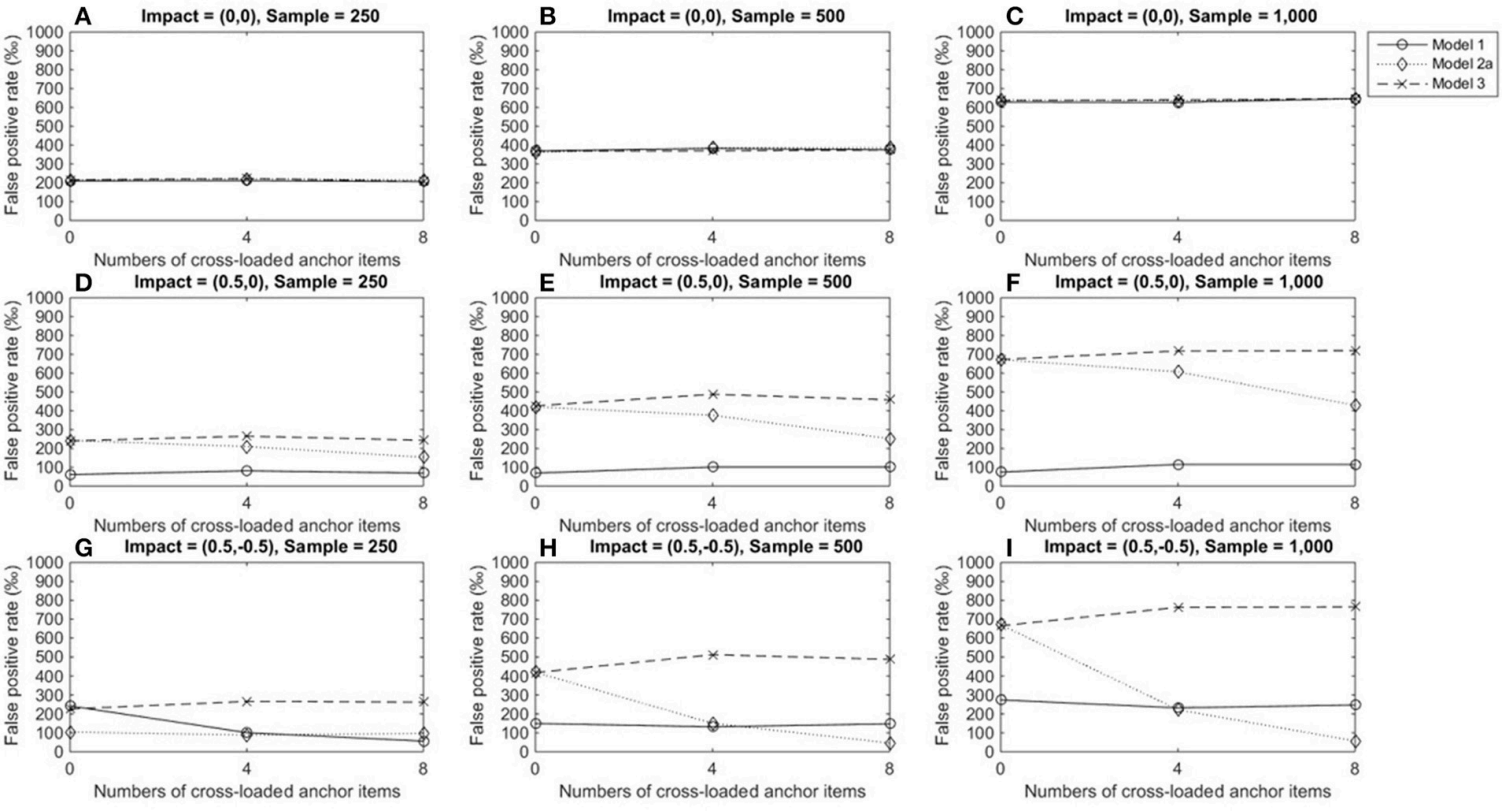
True positive rates when the tested item measured one single domain and DIF magnitude was 0.3. Fore model 1, total scores served as a matching variable; for model 2a, the subset score of domain 1 was used to match participants; model 3 used two subset scores as matching variable. Model 2b was excluded because the tested item did not measure the second domain. **(A)** Impact = (0,0), Sample = 250; **(B)** Impact = (0,0), Sample = 500; **(C)** Impact = (0,0), Sample = 1,000; **(D)** Impact = (0.5,0), Sample = 250; **(E)** Impact = (0.5,0), Sample = 500; **(F)** Impact = (0.5,0), Sample = 1,000; **(G)** Impact = (0.5, −0.5), Sample = 250; **(H)** Impact = (0.5, −0.5), Sample = 500; **(I)** Impact = (0.5, −0.5), Sample = 1,000.

Figure 3 depicts TPRs under medium DIF conditions, when the tested item measured a single dimension. Figures 3A–C show that regardless of test structures and the number of cross-loaded anchor items, the LR approach that incorporated varied matching variables showed similar TPRs when there was no impact (around 0.2 for a group size of 250, 0.4 for a group size of 500, and almost 0.6 for a group size of 1,000). As group sizes increased, TPRs increased. Figures 3D–I indicate that model 3 yielded consistent TPRs and patterns across conditions, and TPRs reached 0.7 at the acceptable level (Cohen and Cohen, 1983) when group sizes were 1,000. Model 1, however, consistently yielded low TPRs (0.1 or below) when there was an impact in the primary domain as well as impacts in both dimensions (TPRs = 0.05–0.27). The performance of model 2 was influenced by impacts and the number of cross-loaded anchor items. When an impact occurred in the primary domain, compared to conditions of no impacts, model 2 yielded slightly decreasing TPRs, as more anchor items measuring both domains were involved. When impacts occurred in both domains, TPRs fell when more anchor items were cross-loaded; performance further deteriorated when 40% of anchor items (8 items) were cross-loaded in the two domains.

**Figure 4:**
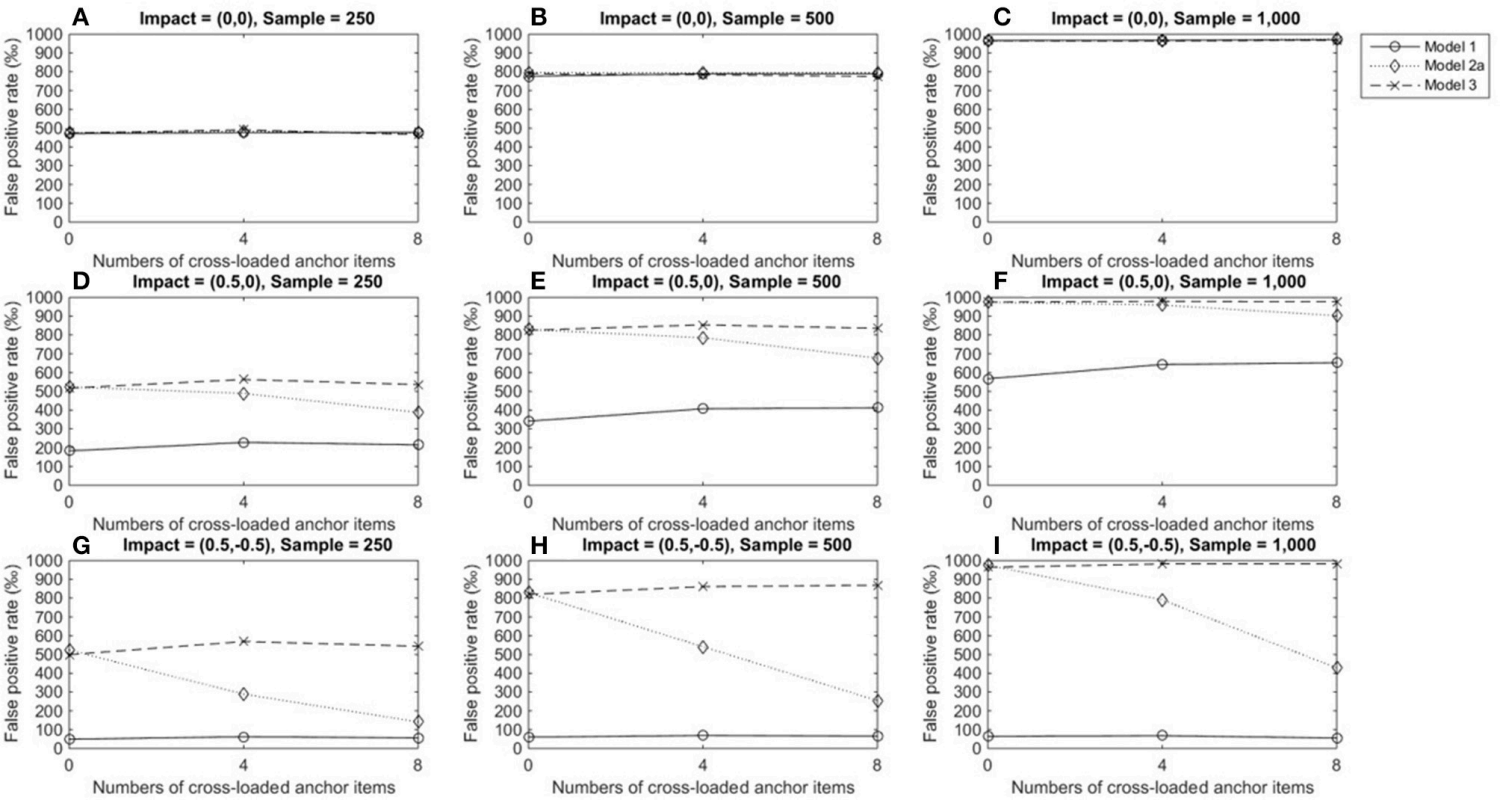
True positive rates when the tested item measured one single domain and DIF magnitude was 0.5. Fore model 1, total score served as a matching variable; for model 2a, the subset score of domain 1 was used to match participants; model 3 used two subset scores as a matching variable. Model 2b was excluded because the tested item did not measure the second domain. **(A)** Impact = (0,0), Sample = 250; **(B)** Impact = (0,0), Sample = 500; **(C)** Impact = (0,0), Sample = 1,000; **(D)** Impact = (0.5,0), Sample = 250; **(E)** Impact = (0.5,0), Sample = 500; **(F)** Impact = (0.5,0), Sample = 1,000; **(G)** Impact = (0.5, −0.5), Sample = 250; **(H)** Impact = (0.5, −0.5), Sample = 500; **(I)** Impact = (0.5, −0.5), Sample = 1,000.

Figures 4A–C show that when there was large DIF and no impact, the three models yielded similar TPRs (around 0.5 for a group size of 250, 0.8 for a group size of 500, and almost 1.0 for a group size of 1,000), and TPRs reached satisfactory levels when group sizes were 500 or 1,000. Figures 4D–I show that the performance of model 3 was not influenced by conditions or impacts and yielded almost identical TPRs as in Figures 4A–C. The performances of models 1 and 2a were influenced by impacts. When an impact occurred in the primary domain, TPRs in model 1 were lower than TPRs when there was no impact; since more anchor items measured more than one domain, TPRs increased slightly (Figures 4D–F) and reached acceptable levels (≈ 0.7) when both group sizes were 1,000. However, TPRs in model 1 fell close to the nominal level when impacts occurred in both domains (Figures 4G–I). Moreover, when impacts occurred, model 1 consistently yielded the lowest TPRs compared to the other two models (Figures 4D–I). Model 2a's performance was related to both impacts and the number of cross-loaded anchor items. As the number of cross-loaded anchor items increased, TPRs decreased but were at acceptable levels when group sizes were 500 or 1,000 and impact occurred in the primary domain. However, TPR became lower when impacts occurred in both the primary and secondary domains, and only when no cross-loaded anchor items and group sizes of 500 or group sizes of 1,000 and cross-loaded anchor items of 4 or fewer, TPR were 0.8 or above.

**Figure 5:**
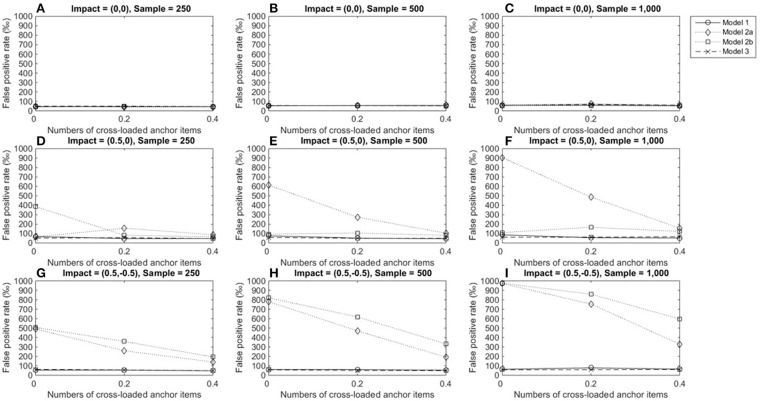
False positive rates when the tested item measured two domains. Fore model 1, total score served as a matching variable; for model 2a, the subset score of domain 1 was used to match participants; for model 2b, the subset score of domain 2 used to match participants; model 3 used two subset scores as a matching variable. **(A)** Impact = (0,0), Sample = 250; **(B)** Impact = (0,0), Sample = 500; **(C)** Impact = (0,0), Sample = 1,000; **(D)** Impact = (0.5,0), Sample = 250; **(E)** Impact = (0.5,0), Sample = 500; **(F)** Impact = (0.5,0), Sample = 1,000; **(G)** Impact = (0.5, −0.5), Sample = 250; **(H)** Impact = (0.5, −0.5), Sample = 500; **(I)** Impact = (0.5, −0.5), Sample = 1,000.

**Figure 6:**
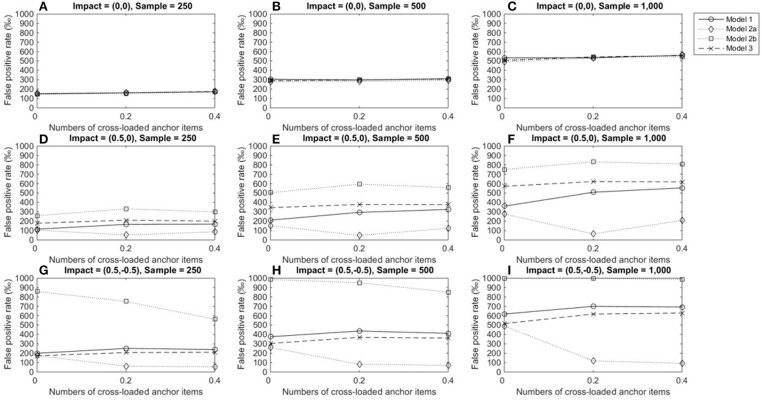
True positive rated when the tested item measured two domain and DIF magnitude was 0.3. For model 1, total scores served as a matching variable; for model 2a, the subset score of domain 1 was used to match participants; for model 2b, the subset score of domain 2 was used to match participants; model 3 used two subset scores as matching variable. **(A)** Impact = (0,0), Sample = 250; **(B)** Impact = (0,0), Sample = 500; **(C)** Impact = (0,0), Sample = 1,000; **(D)** Impact = (0.5,0), Sample = 250; **(E)** Impact = (0.5,0), Sample = 500; **(F)** Impact = (0.5,0), Sample = 1,000; **(G)** Impact = (0.5, −0.5), Sample = 250; **(H)** Impact = (0.5, −0.5), Sample = 500; **(I)** Impact = (0.5, −0.5), Sample = 1,000.

**Figure 7:**
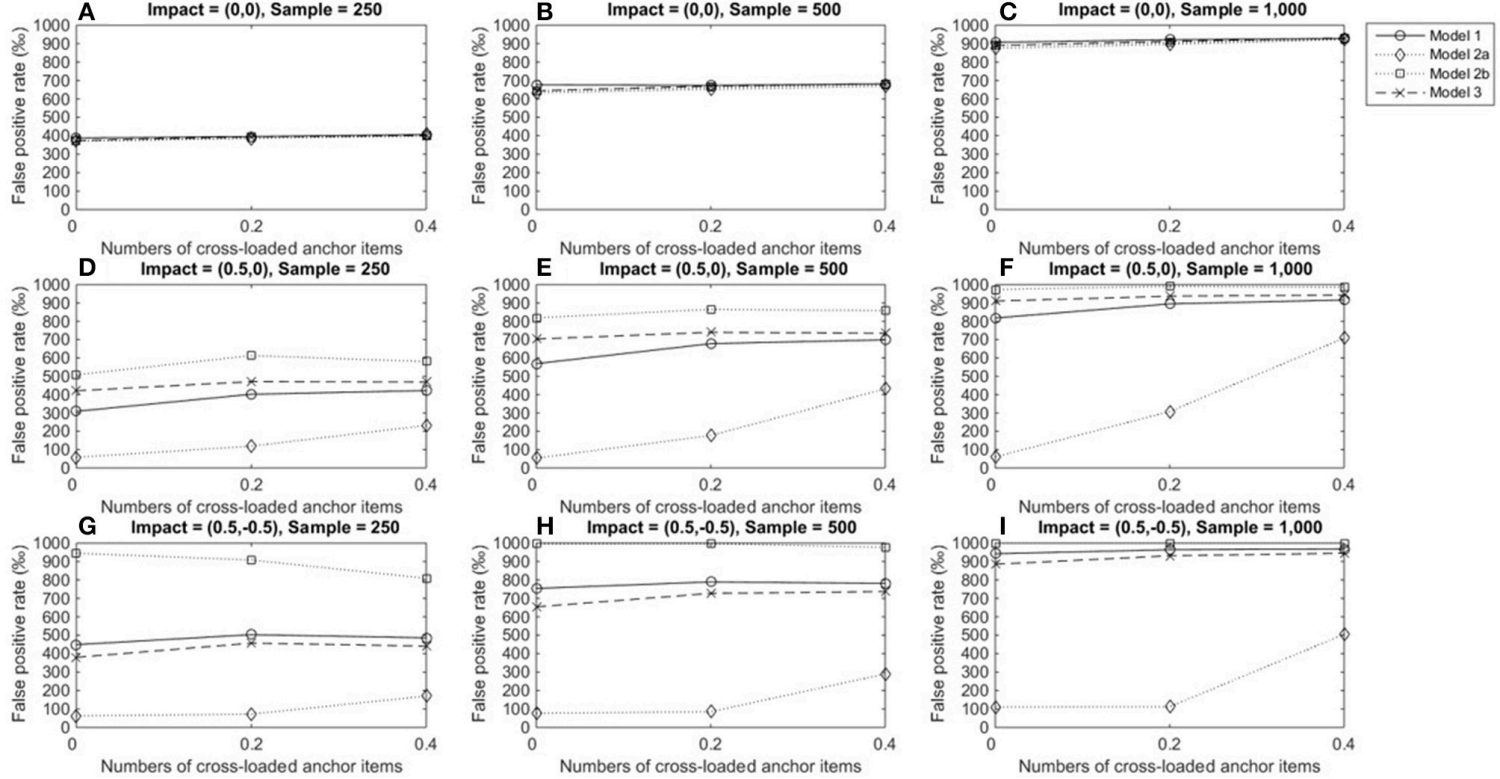
True positive rates when the tested item measured two domains and DIF size was 0.5. For model 1, total scores served as a matching variable; for model 2a, the subtest score of domain 1 was used to match participants; for model 2b, the subset score of domain 2 was used to match participants; model 3 used two subset scores as a matching variable. **(A)** Impact = (0,0), Sample = 250; **(B)** Impact = (0,0), Sample = 500; **(C)** Impact = (0,0), Sample = 1,000; **(D)** Impact = (0.5,0), Sample = 250; **(E)** Impact = (0.5,0), Sample = 500; **(F)** Impact = (0.5,0), Sample = 1,000; **(G)** Impact = (0.5, −0.5), Sample = 250; **(H)** Impact = (0.5, −0.5), Sample = 500; **(I)** Impact = (0.5, −0.5), Sample = 1,000.

Figures 5–**7** describe the performance of four LR procedures under conditions where the item under consideration measured two domains. When no impact occurred, the four models yielded similar FPRs and their performance was not influenced by group size or the number of cross-loaded anchor items (Figures 5A–C). Models 1 and 3 were robust when impacts occurred in domain 1 and showed good control of FPRs when the items under consideration were within-multidimensional (Figures 5D–F). However, once impacts occurred, models 2a and 2b yielded inflated FPRs, particularly when impacts occurred in both the primary and secondary domains (Figures 5G–I). As more cross-loaded anchor items became involved, FPRs diminished but were still far beyond the nominal level of 0.05.

Figure 6 shows TPRs when medium DIF occurred under various conditions. The four models yielded almost identical TPRs when no impact occurred across conditions, and TPRs increased from 0.15 to 0.56 as sample sizes increased (Figures 6A–C). Results also showed that the performances of models 1 and 3 were relatively consistent across impacts and conditions and yielded increasing TPRs as group size increased. When an impact occurred in domain 1, model 3 yielded slightly higher TPRs (≈ 0.18 for sample sizes of 250, ≈ 0.34 for sample sizes of 500, and ≈ 0.57 for sample sizes of 1,000) than model 1 (Figures 6D–F). When impacts occurred in both domains, model 1 yielded slightly higher TPRs (≈ 0.20 for sample sizes of 250, ≈ 0.38 for sample sizes of 500, and ≈ 0.62 for sample sizes of 1,000) than model 3 (Figures 6G–I). The same patterns were found across the number of cross-loaded anchor items, and TPRs were lower than the acceptable level of 0.7. Model 2a consistently yielded the lowest TPRs when impacts occurred, while model 2b yielded the highest TPRs when impacts occurred. Because models 2a and 2b yielded severely inflated FPRs when impacts occurred, TPRs became questionable in these cases.

The above patterns were found in conditions in which large DIF occurred (Figure 7); thus, we have not provided details on those conditions in this paper. One noticeable difference related to the impact of DIF magnitudes is that when DIF sizes were large, both model 1 and 3 yielded TPRs nearly or higher than 0.7 when both group sizes were 500 or 1,000.

If we look at the combined findings under all conditions, model 3 was robust against impacts and conditions and clearly outperformed the other alternatives when scales were designed to be multidimensional. Therefore, we suggest the use of model 3 for DIF detection.

## Discussion, conclusion, and future work

The present study has confirmed the indications from previous research on DIF in multidimensional data: a matching criterion closer to the actual test structure may yield higher power in DIF detection. We have substantially extended the existing evidence on between- and within-item multidimensional data and have further articulated the impact of deviation from unidimensionality of the matching variable as well as various issues of impacts and cross-loadings in latent abilities. The test structures of the matching variable and the item under consideration, group mean differences, and the number of cross-loaded items in the matching variable were manipulated to explore the importance of dimensionality of the matching criterion for DIF assessment. The results have validated existing findings and clarified the impact of multidimensionality on DIF assessment under a variety of conditions, thereby confirming the importance of the dimensionality of a matching variable among different conditions and demonstrating that the matching criterion that best reflected the test structure performed consistently well across all conditions.

These simulation studies have suggested that once the matching variable accurately reflects the underlying multidimensionality of a scale (e.g., model 3), the model will yield satisfactory FPRs and more accurate TPRs. Other alternative models that incorporated total scores or a subdomain score yielded severely inflated FPRs and questionable TPRs when impacts were involved and interacted with multidimensionality. The suggested model (model 3) that incorporated multiple subdomain scores was also robust against the impact of differences in group mean ability and test structure. Compared to conventional LR approaches, the proposed model consistently yielded similarly, more satisfactory findings across conditions—either between-item or within-item multidimensionality—regardless of whether one or more impacts had occurred.

Our findings also indicate that when no impacts were involved, all models yielded satisfactory FPRs across all conditions, regardless of the number of cross-loaded anchor items or the number of dimensions measured by the item under consideration. The dimensionality of a matching variable did not lead to inflated or deflated FPRs, as suggested in previous studies (e.g., Mazor et al., 1995; Clauser et al., 1996). One possibility for this discrepancy is the differing approaches to subtest score estimation. Previous studies have treated cross-loaded items as non-cross-loaded in DIF detection and have ignored the impact of cross-loaded items in the use of multiple subtest scores as a matching variable. Consequently, the matching criterion does not reflect the test's true underlying factor structure. In the present study, the matching variable using two subtest scores combined cross-loaded and non-cross-loaded items. Responses to all cross-loaded items provided information about both domains; these responses were combined with non-cross-loaded items to calculate individual subtest scores. This method may more accurately reflect the underlying test structure. Thus, the evidence from the present study appears stronger than that from other studies.

When an item under consideration was between-item multidimensional and when impacts occurred, the dimensionality of the matching criterion showed impacts on the assessment of DIF detection. A matching variable that approximated the test structure outperformed the other models across all conditions, particularly when impacts occurred in both the primary and secondary domains, where the reference group had a higher mean ability in the primary domain and lower mean ability in the secondary domain compared to the focal group. The performance improvement when using multiple subtest scores was profound. These findings also suggest that the conventional LR approach (using total scores as a matching variable) was the poorest performer and yielded inflated FPRs and the lowest TPRs compared to other models. The conventional LR procedure failed to accurately detect DIF items in between-item multidimensional data when impacts occurred.

Consistent with previous studies (e.g., Li, 2014), the magnitudes of DIF and group sizes showed impacts on TPRs when LR was used in DIF assessments. The proposed model yielded satisfactory TPRs (≥0.7) when group sizes were 1,000 regardless of the magnitudes of DIF, or when DIF sizes were large and group sizes were 500 or larger. In other words, even though the proposed model in general outperformed other alternatives, it could not yield satisfactory TPRs of 0.7 when both DIF sizes and group sizes were small. Researchers or practitioners need to recruit enough sample sizes in order to achieve a desirable power when implementing the proposed approach to detect DIF in multidimensional data.

The impact of multidimensionality on the detection of non-uniform DIF was not investigated in the present study; it is worthy of being studied in future research. The present study and previous studies (e.g., Yao and Li, 2015) have confirmed that using a unidimensional approach would result in unequal expected item scores for different groups, leading biased conclusions in DIF assessment. We can foresee that the performance of LR in detecting non-uniform DIF will be influenced by the multidimensional structure as well. It must be noted that when investigating non-uniform DIF on an item measuring *D* constructs, there are a total of 3^*D*^ possible outcomes. For example, take a two-dimensional item including two slope parameters, as in Equation (5). Consequently, there will be nine (= 3^2^) possible outcomes in non-uniform DIF detections: (1) both the discriminations are identical for the two groups; (2) both the discriminations are higher for the focal group; (3) both the discriminations are higher for the reference group; (4) only the first discrimination is higher for the focal group; (5) only the first discrimination is higher for the reference group; (6) only the second discrimination is higher for the focal group; (7) only the second discrimination is higher for the reference group; (8) the first discrimination is higher for the focal group, whereas the second discrimination is higher for the reference group; and (9) the first discrimination is higher for the reference group, whereas the second discrimination is higher for the focal group. A two-dimensional item would be flagged as non-uniform DIF in all but the first outcomes. A more dedicated simulation study is required to uncover how the multidimensionality could lead to non-uniform DIF.

While the present study has comprehensively examined the influence of several factors on the performance of the LR procedure for uniform DIF detection in multidimensional data, further investigations are still warranted. First, an identical correlation between two latent variables for the two groups was assumed in our simulations. The extent of the influence of unequal correlations on the difference of item scores has not been studied yet. Second, the power of the LR approach is undermined by the percentage of DIF items in the matching variable. Future studies should manipulate the percentage of DIF items in the anchor variable to examine the influence of interactions on DIF detection in multidimensional data. Finally, the present study evaluated only the performance of the LR procedure; future studies must compare LR performance with the performance of the MH, SIBTEST, and multidimensional multi-group IRT (e.g., Yao and Li, 2010, 2015) in both simple and complex structured data to provide further insights for practical use.

## Author contributions

H-FC contributed to the conception, design, and analysis of data as well as drafting and revising the manuscript. K-YJ contributed to the design and critically revising the manuscript.

### Conflict of interest statement

The authors declare that the research was conducted in the absence of any commercial or financial relationships that could be construed as a potential conflict of interest.
